# Mutations in the Rice *OsCHR4* Gene, Encoding a CHD3 Family Chromatin Remodeler, Induce Narrow and Rolled Leaves with Increased Cuticular Wax

**DOI:** 10.3390/ijms20102567

**Published:** 2019-05-25

**Authors:** Tingting Guo, Daofeng Wang, Jingjing Fang, Jinfeng Zhao, Shoujiang Yuan, Langtao Xiao, Xueyong Li

**Affiliations:** 1 Hunan Provincial Key Laboratory of Phytohormones, Hunan Provincial Key Laboratory for Crop Germplasm Innovation and Utilization, College of Bioscience and Biotechnology, Hunan Agricultural University, Changsha 410128, China; guoting0118@126.com; 2 National Key Facility for Crop Gene Resources and Genetic Improvement, Institute of Crop Science, Chinese Academy of Agricultural Sciences, Beijing 100081, China; w2806047078@126.com (D.W.); fangjingjing@caas.cn (J.F.); zhaojinfeng@caas.cn (J.Z.); 3 Shandong Rice Research Institute, Jinan 250100, China; ysj868@sina.com

**Keywords:** auxin, chromatin remodeling factor, cuticular wax, drought tolerance, epigenetic regulation, leaf width, histone modification, narrow leaf, *OsCHR4*, rice

## Abstract

Leaf blade width, curvature, and cuticular wax are important agronomic traits of rice. Here, we report the rice *Oschr4-5* mutant characterized by pleiotropic phenotypes, including narrow and rolled leaves, enhanced cuticular wax deposition and reduced plant height and tiller number. The reduced leaf width is caused by a reduced number of longitudinal veins and increased auxin content. The cuticular wax content was significantly higher in the *Oschr4-5* mutant, resulting in reduced water loss rate and enhanced drought tolerance. Molecular characterization reveals that a single-base deletion results in a frame-shift mutation from the second chromodomain of OsCHR4, a CHD3 (chromodomain helicase DNA-binding) family chromatin remodeler, in the *Oschr4-5* mutant. Expressions of seven wax biosynthesis genes (*GL1-4*, *WSL4*, *OsCER7*, *LACS2*, *LACS7*, *ROC4* and *BDG*) and four auxin biosynthesis genes (*YUC2*, *YUC3*, *YUC5* and *YUC6*) was up-regulated in the *Oschr4-5* mutant. Chromatin immunoprecipitation assays revealed that the transcriptionally active histone modification H3K4me3 was increased, whereas the repressive H3K27me3 was reduced in the upregulated genes in the *Oschr4-5* mutant. Therefore, *OsCHR4* regulates leaf morphogenesis and cuticle wax formation by epigenetic modulation of auxin and wax biosynthetic genes expression.

## 1. Introduction

In plants, leaves are the major organ used for photosynthesis and transpiration, and are considered to be the main source of dry matter accumulation in plants. Blade width and curvature are key factors of leaf morphology. As reported, blade width, particularly that of the first and second uppermost leaves, can significantly influence the rice grain yield, whereas appropriately rolled leaves can optimize canopy light transmission, increase the effective leaf area per unit of land and, consequently, the canopy photosynthesis efficiency [1]. 

To date, a number of narrow and/or rolled leaf genes have been reported in rice. The majority of narrow leaf genes are related to auxin signaling. For example, a mutant of *Nal1* (encoding a plant-specific protein with unknown biochemical function) exhibited a characteristic phenotype of narrow leaves due to decreased number of longitudinal veins and polar auxin transport capacity [2,3]. Mutation in *TDD1* (encoding a protein homologous to anthranilate synthase beta-subunit involved in the upstream of Trp-dependent auxin biosynthesis) could induce obviously narrow leaves [4]. *NAL7* and its allelic gene *CONSTITUTIVELY WILTED1* encode an auxin biosynthesis enzyme OsYUCCA8 and their mutants exhibit narrow leaves [5,6]. Similar to the auxin-deficient mutant, the leaves of the *osarf11-1*, a loss-of-function mutant of the *auxin-response factor 11* (*OsARF11*), are also moderately narrowed [7]. Mutations in genes involved in other pathways could also produce narrow leaves such as *NAL9* (encoding an ATP-dependent Clp protease proteolytic subunit) and *NAL11* (encoding a heat shock DNAJ protein) [8,9]. For rolled leave, it could be divided into adaxialized and abaxialized curling. The mutant of *SLL1*/*RL9* (encoding a SHAQKYF class MYB family transcription factor) displayed completely adaxialized leaves [10]. This gene mainly functions in the modulation of sclerenchymatous cells formation on the abaxial side during leaf development [11]. The mutant of *ADL1* (a plant-specific calpain-like cysteine proteinase) exhibited abaxially rolled leaves due to abnormal bulliform-like cells distribution [12]. Meanwhile, overexpression of *ACL1* (encodes a protein with unknown functional domains) and its homolog *ACL2* caused abaxial leaf curling [13], while overexpression of *OsAGO7* was correlated with adaxial leaf curling [14]. *SRL1* encodes a putative glycosylphosphatidylinositol-anchored protein and modulates leaf adaxial rolling by regulating the formation of bulliform cells [15]. Interestingly, several genes were associated with both narrow and rolled leaves. The mutants of *NRL1* (encoding cellulose synthase-like protein D4), *NRL2* and *NRL3* (both encoding a novel protein with unknown biochemical function) could induce semi-rolled leaves with reduced blade width [16,17,18,19].

The surface of rice leaf is covered by cuticular wax which provides self-protection against potential external stresses, such as non-stomatal water loss, pathogen infection and ultraviolet (UV) radiation [20]. The main composition of cuticular wax is the very-long-chain fatty acids (VLCFAs) as well as their following derivatives: aldehydes, primary and secondary alcohols, alkanes, ketones and wax esters. Through years of study, many genes encoding enzymes and transcription factors involved in biosynthesis of cuticular wax in rice have been uncovered by bioinformatics and genetic approaches. The *Glossy1* (*GL1*) gene family, reported first in maize, encodes the fatty aldehyde decarbonylase required for the synthesis of cuticular wax. Through homologous sequence alignment, 11 *OsGL1* family genes were found in rice [21]. Subsequent studies validated that *OsGL1-1*/*WSL2*, *OsGL1-2*, *OsGL1-3*, *OsGL1-5*/*Wda1* and *OsGL1-6* affect wax synthesis on various parts of rice plant [21,22,23,24,25]. AP2/ERF family transcription factors OsWR1 and OsWR2, homologs of the *Arabidopsis WIN1/SHN1* gene, promote wax production via activating wax biosynthesis gene [26,27]. Homeodomain-leucine zipper class IV family transcription factor ROC4 positively regulates cuticular wax biosynthesis via directly binding to the conserved L1 box *cis*-element in the promoters of Os-*BDG*, which encodes an extracellular synthase responsible for the formation of cuticle [28]. DROUGHT HYPERSENSITIVE (DHS), a RING-type E3 ligase, interacts with ROC4 and promotes its degradation via the ubiquitin/26S proteasome [28]. After synthesized in the endoplasmic reticulum, wax is transferred to the plant surface via adenosine triphosphate binding cassette transporters as well as lipid transfer proteins [29].

Proper regulation of gene expression during development is dependent on the dynamic modifications of chromatin, during which the accessibility of specific DNA regions to the transcription machinery may be altered. The chromodomain helicase DNA-binding (CHD) proteins are one family of chromatin remodeling factors, which are defined by a plant homeodomain (PHD) finger, two chromodomains, one SNF2 (sucrose non-fermenting)-related ATPase/helicase domain, and one DNA binding motif [30,31]. CHD chromatin remodelers directly change the chromatin structures and are important regulators of gene expression. Mutations of CHD protein could always induce pleiotropic phenotypes in plants. PICKLE is a typical CHD protein in *Arabidopsis*. The *pickle* mutant was initially characterized by pickle roots with green tuber due to failed repression of seed-specific genes [32]. This protein was then found to function in repression of ectopic stipules and meristems in leaf tissue and inhibition of meristematic genes in carpel tissue [33,34]. In rice, the closest homolog with PICKLE is OsCHR702, while both T-DNA insertion mutation and RNAi of *OsCHR702* did not produce any morphological phenotypes [35,36]. OsCHR4 is another CHD member characterized in rice. The *Oschr4* mutant shows defective chloroplasts in the adaxial mesophyll cells due to blocked proplastid growth and thylakoid membrane formation [37]. As an allelic gene of *OsCHR4*, *OsCHR729* was revealed to encode a bifunctional chromatin regulator associated with gene transcription regulation through modulation of histone H3K4 and H3K27 methylation during plant development [36]. Meanwhile, the Os*chr729* mutant induced by T-DNA insertion shows pleiotropic phenotypes, including small and rolled leaves, reduced stem elongation and chlorophyll contents, and absence of secondary panicle branches [36]. One study reported that *CHR729* regulates seedling development by affecting contents of gibberellin acid (GA) in rice, and the transcript level of GA biosynthesis genes was altered in the corresponding mutant line *t483* [38]. In addition, *CRL6*, an allelic gene of *CHR729*, was speculated to regulate crown root development via auxin signaling pathway due to decreased expression of most *OsIAA* genes in *crl6* compared with the wild type (WT) [39]. Recently, *rfs,* another allelic mutant of *OsCHR4/OsCHR729*, was characterized by the chlorotic and cell death phenotypes due to accumulation of reactive oxygen species (ROS). Both transcript and H3K4me3 levels of five ROS-related genes declined in the *rfs* mutant [40].

In this study, three additional allelic mutants of *OsCHR4* were reported, namely *Oschr4-5*, *Oschr4-6*, and *Oschr4-7*, which exhibited pleiotropic phenotypes such as narrow and rolled leaf, reduced plant height, tiller number and seed size at mature stage. Anatomical investigations revealed that the reduced leaf width was due to a decreased number of major and minor veins while the cell size remained unchanged. The auxin content was significantly higher in *Oschr4-5*, which may explain the altered venation pattern and leaf size at physiological level. Interestingly, more cuticular wax was deposited on the surface of the *Oschr4-5* leaves, which reduced the water-loss rate and enhanced the drought tolerance. Expression levels of several auxin and wax biosynthesis genes were up-regulated in the *Oschr4-5* mutant. Consistent with the role of CHD3 protein in chromatin remodeling, histone modifications H3K4me3 and H3K27me3 in the upregulated genes were increased and reduced in *Oschr4-5,* respectively. Our data reveal novel functions of *OsCHR4* in the epigenetic regulation of auxin and wax biosynthesis-related genes, leaf development and cuticle wax formation in rice.

## 2. Results

### 2.1. Pleiotropic Phenotype of the Oschr4-5 Mutant

**Figure 1:**
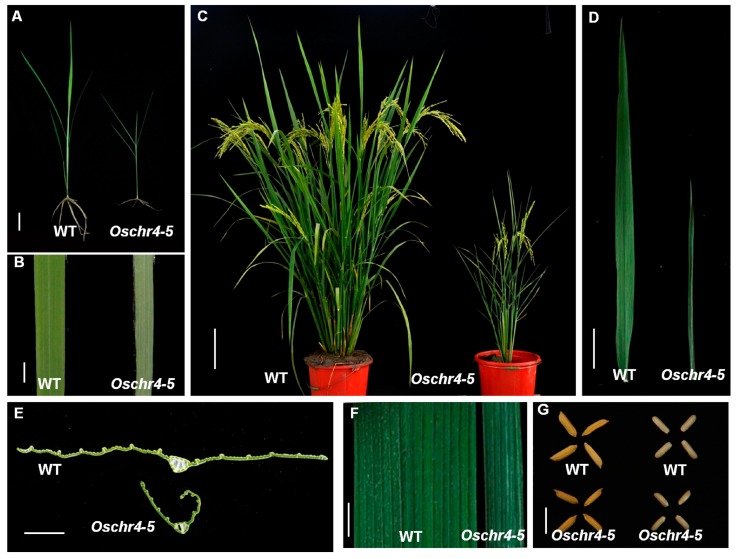
Phenotypic characterization of the *Oschr4-5* mutant. (**A**) Phenotypes of wild type (WT) and *Oschr4-5* mutant plants at seedling stage. Bar = 1 cm. (**B**) Adaxial side of leaf blade from wild type and *Oschr4-5* mutant at seedling stage. Bar = 0.5 cm. (**C**) Phenotypes of wild type and *Oschr4-5* mutant plants at mature stage. Bar = 15 cm. (**D**) Comparison of leaf length between wild type and *Oschr4-5* mutant at mature stage. Bar = 5 cm. (**E**) Cross sections through mature leaves from wild type and *Oschr4-5* mutant. Bar = 0.25 cm. (**F**) Comparison of leaf width between wild type and *Oschr4-5* mutant at mature stage. Bar = 0.5 cm. (**G**) Phenotypes of seeds from wild type and *Oschr4-5* mutant. Bar = 1 cm.

A rice mutant characterized by narrow and rolled leaves was isolated from the gamma ray mutagenized *Indica* rice cultivar Indica 9, which was named as *Oschr4-5* based on the gene identity revealed in the following study. At the seedling stage, slight dwarfism and albino on the adaxial side of leaves were observed in *Oschr4-5* (Figure 1A,B). At the mature stage, the plant height and tiller number of *Oschr4-5* was significantly reduced by 45% and 76% when compared with that of WT (*p* < 0.01) (Figure 1C, Table S1). The length and width of the leaves in *Oschr4-5* was significantly reduced by 38% and 50% when compared with that of the WT (*p* < 0.01) (Figure 1D,F, Table S1). Meanwhile, smaller seeds were also observed in *Oschr4-5* (Figure 1G). Later on, two allelic mutants, namely *Oschr4-6* and*Oschr4-7*, were isolated from the Ethyl methane sulfonate (EMS) mutagenized *japonica* cultivar Nipponbare, which exhibited the same pleiotropic phenotypes, including narrow and rolled leaves with albino on the adaxial side, significantly reduced leaf length, plant height, tiller number and seed size (Figure S1, Table S1).

### 2.2. Anatomical and Physiological Basis of Reduced Leaf Width

**Figure 2:**
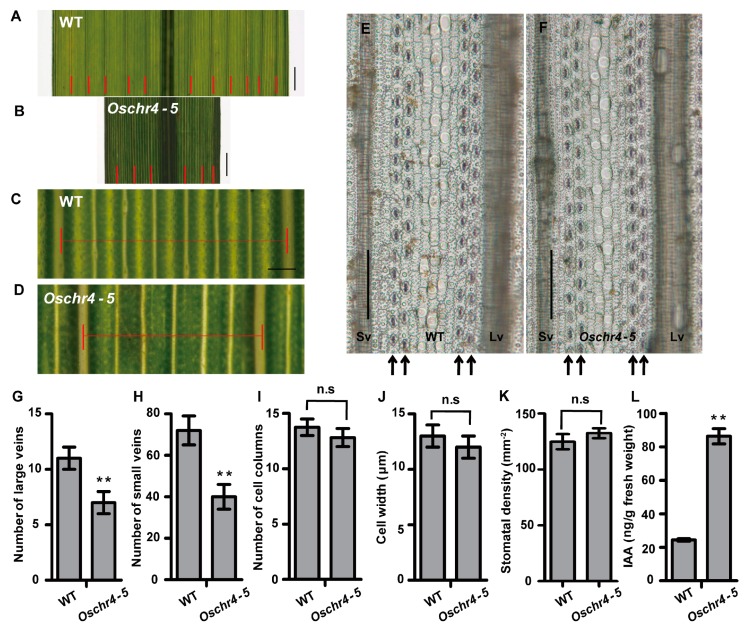
The *Oschr4-5* mutant affects leaf width. (**A**–**D**) The morphology of leaf veins from wild type and *Oschr4-5*mutant. Red bars represent large veins. Bar = 0.2 cm (**A**,**B**), 0.2 mm (**C**,**D**). (**E**,**F**) The abaxial epidermal cells of leaf blade from wild type and *Oschr4-5* mutant. Bar = 50 μm. The columns with stoma were indicated with black arrow. Sv: Small vein; Lv: Large vein. (**G**–**K**) Comparison of the large veins (**G**), small veins (**H**), cell columns (**I**), cell width (**J**) and stomatal density (**K**) on abaxial epidermis of leaf blade and indole acetic acid (IAA) contents between wild type and *Oschr4-5* mutant (**L**). Data are the means ± standard errors, *n* = 15 (**G**–**J**), 5 (**K**), 3 (**L**). Significance of data is tested by student’s t test. ** *p* < 0.01.

To reveal the underlying cellular basis of narrow leaves in the *Oschr4-5* mutant, the venation pattern of leaf was first observed. The number of large veins and small veins of *Oschr4-5* was significantly reduced by 36% and 42%, respectively, of that of the WT (*p* < 0.01) (Figure 2A–D,G–H). Then the epidermal cell profile on the abaxial side of leaf blade was examined. Neither the number of cell column between the adjacent small vein nor the cell width at the horizontal orientation showed significant difference between *Oschr4-5* and WT (Figure 2E,F,I,J). These results indicate that the decreased leaf width in *Oschr4-5* may be caused by reduction in the number of veins. Since auxin is required for specification of the venation pattern in leaves [3,41] and auxin overproduction usually leads to narrow and epinastic leaves in *Arabidopsis* [42,43], the amount of free indole acetic acid (IAA), the major auxin, in *Oschr4-5* and WT was measured. As a result, the content of IAA in *Oschr4-5* was significantly higher than that of WT (86.39 ± 4.55 vs. 24.39 ± 0.76 ng/g, *p* < 0.01) (Figure 2L).

### 2.3. Increased Cuticular Wax on the Surface of Leaves with Enhanced Drought Tolerance in Oschr4-5

**Figure 3:**
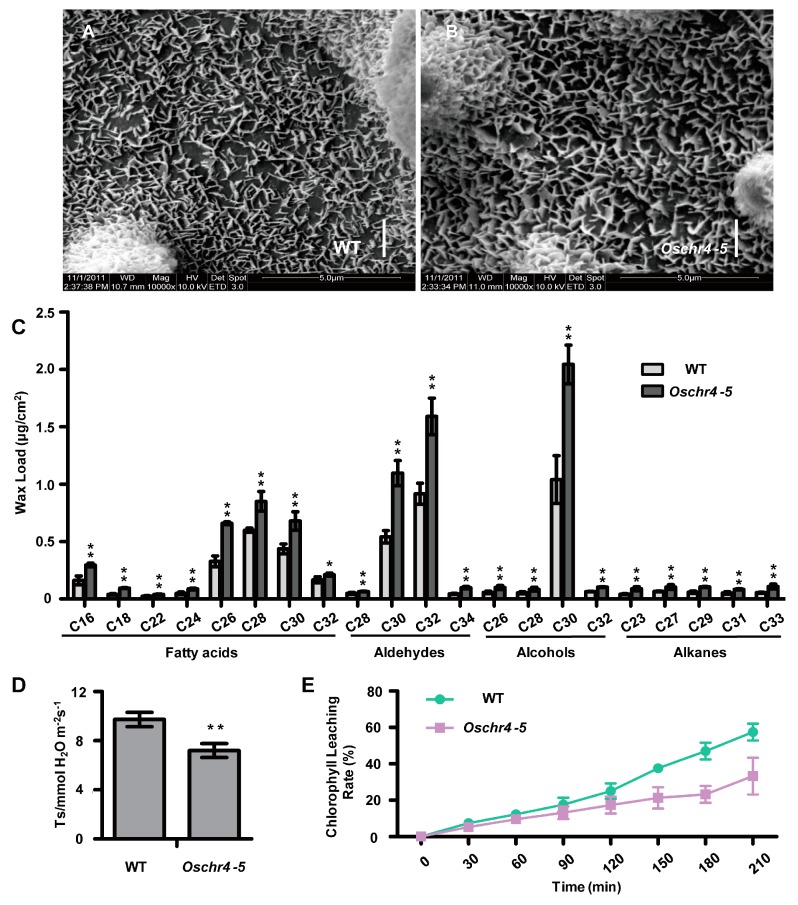
More cuticular wax deposited on leaves of the *Oschr4-5* mutant. (**A**–**C**) Scanning electron microscope (SEM) images of adaxially leaf surface (**A**,**B**) and cuticular wax composition and loads (**C**) from wild type and the *Oschr4-5* mutant. Bar = 2 μm. Data are the means ± standard errors (*n* = 3). Significance of data is tested by student’s *t* test. * *p* < 0.05, ** *p* < 0.01. (**D**,**E**) The transpiration rate (**D**) and chlorophyll leaching rate (**E**) of leaf blades from wild type and *Oschr4-5* mutant. Data are the means ± standard errors (*n* = 3). Significance of data is tested by student’s *t* test. ** *p* < 0.01.

Scanning electron microscopy was used to examine the surface of leaves. Interestingly, the platelet-like wax crystals deposited on the leaves’ surface were larger and denser in *Oschr4-5* compared with WT (Figure 3A,B). Then the compositions and contents of cuticular wax were measured by gas chromatography-mass spectrometry (GC-MS). As shown in Figure 3C, the wax components of fatty acids, aldehydes, alcohols and alkanes were all significantly increased in *Oschr4-5* than WT (*p* < 0.05).

**Figure 4:**
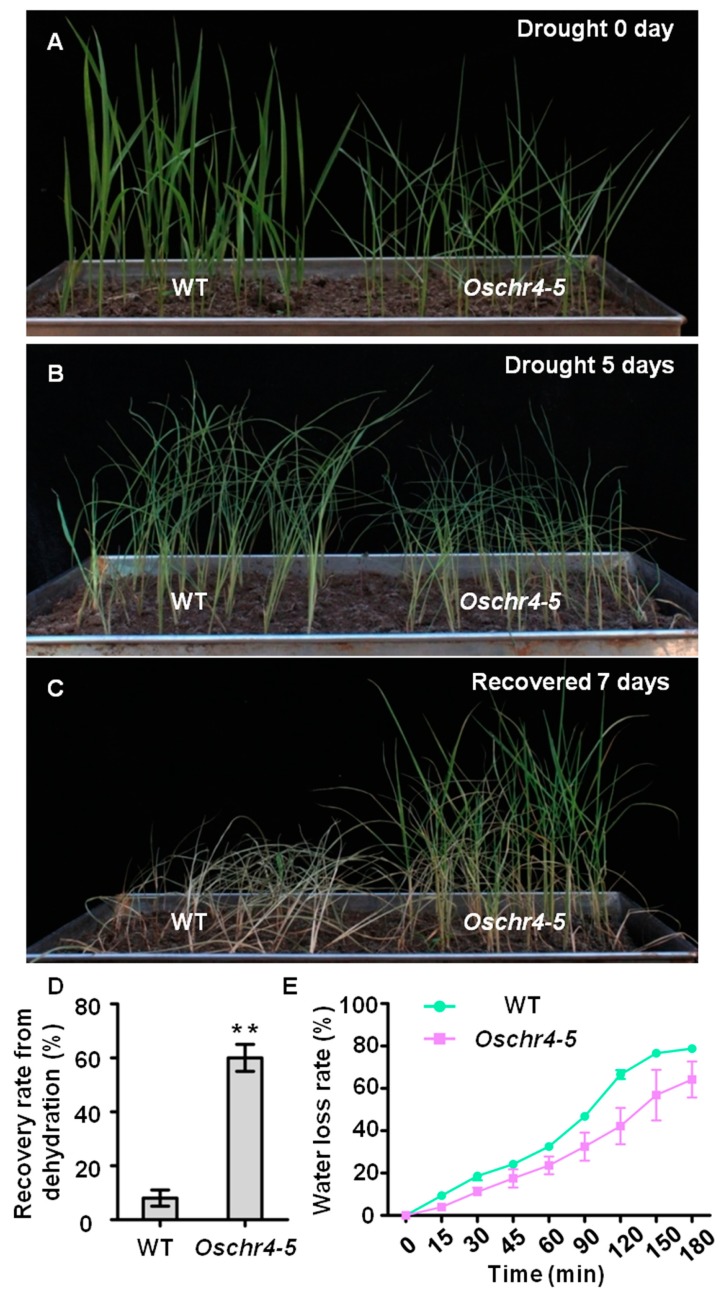
The drought stress responses of the *Oschr4-5* mutant. (**A**–**C**) Drought experiment with the 4 week-old plants for 0 day (**A**), 5 days (**B**), seedling recovered 7 days after rehydration (**C**). (**D**) Recovery rate from dehydration of wild type and *Oschr4-5* mutant in (**C**). Data are the means ± standard errors (*n* = 3). Significance of data is tested by student’s *t* test, ** *p* < 0.01. (**E**) Water loss rate of detached leaf blades between wild type and the *Oschr4-5* mutant. Data are the means ± standard errors (*n* = 3).

As the content and structure of cuticular wax could strongly influence cuticle permeability, its effects on transpiration rate were first evaluated. Indeed, the transpiration rate of *Oschr4-5* was significantly lower than that of WT (*p* < 0.01) (Figure 3D). Secondly, measurement of cuticle membrane permeability as demonstrated by chlorophyll-leaching rate showed that chlorophyll leaches slower from *Oschr4-5* than WT at different time points (Figure 3E). Furthermore, a dehydration-rehydartion treatment revealed that drought tolerance of *Oschr4-5* was significantly enhanced. The *Oschr4-5* seedlings showed significantly higher recovery rate than WT (60% vs. 8%, *p* < 0.01) (Figure 4A–D). The water loss rate was also measured to evaluate the water retention capacity of detached leaves. The data showed that water loss rates of *Oschr4-5* were significantly slower than in WT (Figure 4E). The possible effects of stomatal density and leaf thickness on the differential water loss rate might be ruled out because no significant difference was detected in the stomatal density (Figure 2E,F,K) and leaf blade thickness (WT: 107.43 ± 7.51 μm; *Oschr4-5*: 108.17 ± 7.23 μm) between *Oschr4-5* and WT. These results prove that *Oschr4-5* exhibited lower cuticle permeability and water loss because of higher cuticular wax content on the surface of leaves compared to WT.

### 2.4. Characterization of the Molecular Lesions in the Oschr4 Mutant

**Figure 5:**
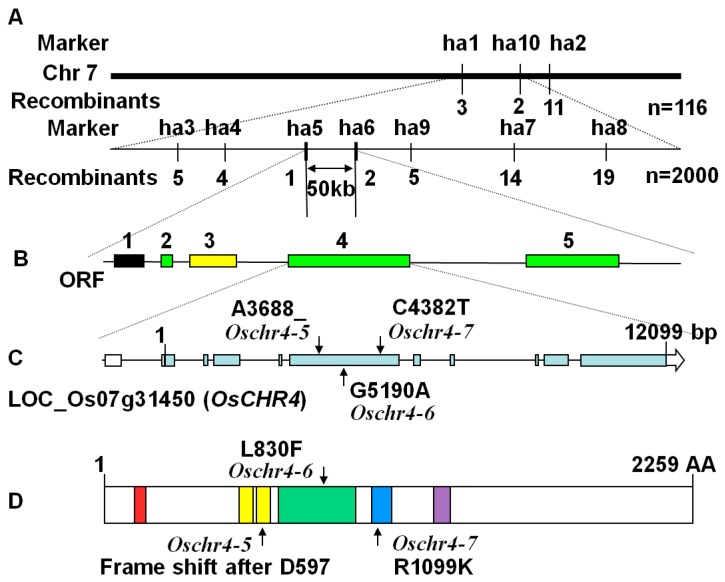
Molecular characterization of mutation sites in the *OsCHR4* gene. (**A**) Fine mapping of the gene on chromosome 7. The mutated gene was localized within 50 kb region delimited by two indel marker ha5 and ha6. (**B**) Predicted open reading frames (ORFs) within the fine mapping interval. Green, ORFs with known biochemical functions; Yellow, ORFs encoding expressed hypothetical proteins; Black, ORFs encoding transposons. (**C**) Gene structure of *OsCHR4*. The mutation sites in the*Oschr4-5*, *Oschr4-6* and *Oschr4-7* mutant were indicated as black arrows. Blue boxes, exons; white boxes, UTR; black line, introns. (**D**) Protein structure of OsCHR4. Each domain is represented in different color box. Red, PHD domain; yellow, Chromo domains; green, SNF2 related helicase/ATPase domain; blue, Helicase C domain; purple, DNA-binding domain.

To find out the molecular lesion responsible for the pleiotropic phenotypes of the *Oschr4-5* mutant, three F_2_ populations were generated (*Oschr4-5* × 9311; *Oschr4-5* × Nipponbare; *Oschr4-5* × 02428). Within these F_2_ populations, a mendelian segregation ratio of 3:1 (χ^2^_0.05_ < 3.84) was calculated, which indicated the mutant phenotype was controlled by a single recessive gene. Then a large F_2_ population generated by *Oschr4-5* × 02428 was used to map the causative locus. In primary mapping, 116 F_2_ mutant individuals located this gene between two Indel markers ha1 and ha10 on the long arm of chromosome 7. Fine mapping using 7 new Indel markers and 2000 F_2_ recessive individuals further localized this gene within 50 kb region delimited by Indel markers ha5 and ha6 (Figure 5A).

According to the MSU (Michigan State University) Rice Genome Annotation Release 7 (http://rice.plantbiology.msu.edu), a total of 5 predicted open reading frames (ORFs) were revealed within the 50 kb fine mapping interval, including 3 ORFs with known biochemical functions (ORF 2: phosphate-induced protein 1; ORF 4: OsCHR4/CHR729; ORF 5: peptide-Nasparagine amidase), 1 ORF encoding expressed hypothetical protein (ORF 3) and 1 transposon (ORF 1) (Figure 5B). Then genomic DNA of all these five ORFs in the *Oschr4-5* mutant was sequenced and compared with that of WT. A single-base deletion at position 3688 was found in the ORF4 in *Oschr4-5* (created through gamma ray radiation), whereas no mutation was found in other four ORFs. Sequence analysis of other two allelic mutants *Oschr4-6* and *Oschr4-7* created through EMS mutagenesis revealed a C4382T and G5190A substitution in ORF4, respectively (Figure 5C). Therefore, ORF4 encoding OsCHR4/CHR729 was found to be the target gene, which is quite large with 11 exons and 10 introns.

### 2.5. Alignment and Phylogenetic Analysis

*OsCHR4* encodes a member of the CHD3 subfamily of chromatin remodeling factors [36,37]. OsCHR4 has 2259 amino acids in length with several putative domains, including one PHD domain, two chromodomains, one SNF2 related helicase/ATPase domain, one Helicase C and one DNA-binding domain from amino terminus to carboxyl terminus (Figure 5D). In the *Oschr4-5* mutant, the single-base deletion results in a frame-shift mutation from the second chromodomain. In *Oschr4-6*, the amino acid substitution L830F (CTC to TTC) occurred within the SNF2 related helicase/ATPase domain. In *Oschr4-7,* the amino acid substitution R1099K (AGA to AAA) occurred within the Helicase C domain (Figure 5D).

Multiple sequence alignment of the OsCHR4 homologous proteins in eight species was performed. As shown in Figure S2A, the substitution mutation sites in *Oschr4-6* and *Oschr4-7* were highly conserved in these species, which highlights the important role of these residuals in CHR4. Then the evolutionary relationship among CHD families in rice and *Arabidopsis* was evaluated. As a result, CHR705 was revealed to belong to the member of subfamily I (CHD1), and the other CHR proteins belonged to subfamily II (CHD3). There were no subfamily III members found in rice or *Arabidopsis*. Meanwhile, PICKLE in *Arabidopsis* was found to be close to CHR702 in rice, and the closest gene with *OsCHR4* in *Arabidopsis* was CHR4 (PKR1) (Figure S2B).

### 2.6. Expression of Several Wax and Auxin Biosynthesis Related Genes Were Upregulated in Oschr4-5

**Figure 6:**
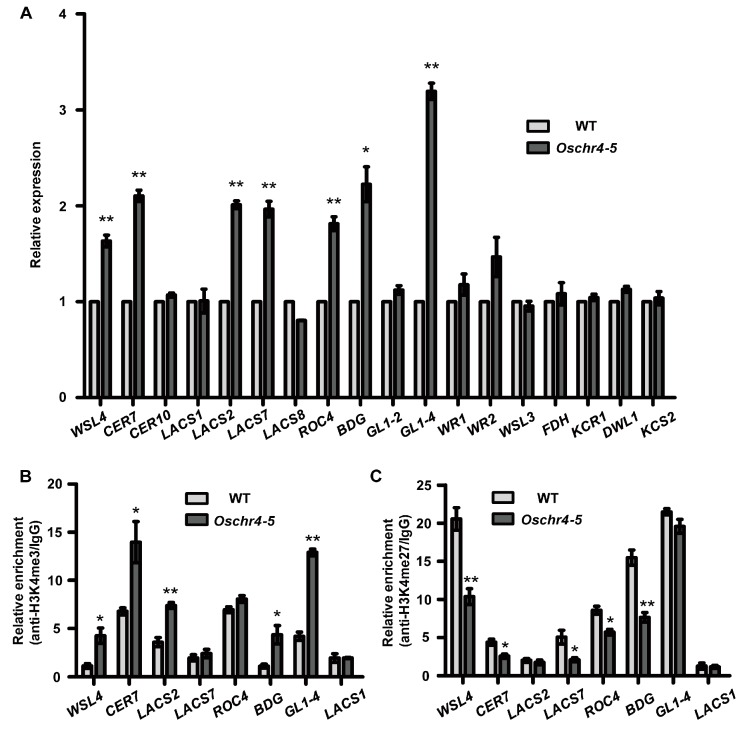
*OsCHR4* regulates expression of genes related to wax biosynthesis. (**A**) Quantitative real-time polymerase chain reaction (qRT-PCR) analysis of 19 wax genes in 30-day-old plants of WT and *Oschr4-5*. Data are the means ± standard errors (*n* = 3). Significance of data is tested by student’s *t* test. * *p* < 0.05, ** *p* < 0.01. (**B**,**C**) ChIP-qPCR analysis for H3K4me3 (**B**) and H3K27me3 (**C**) levels on 7 up-regulated wax genes in *Oschr4-5* relative to the WT. Data are the means ± standard errors (*n* = 3). Significance of data is tested by student’s *t* test. * *p* < 0.05, ** *p* < 0.01.

As a chromatin remodeling factor, the loss of OsCHR4 function was identified to influence the rice plant growth and development in various ways [36,37,38,39,40], but the underlying molecular mechanisms need to be further analyzed. To understand why the content of cuticular wax increased in the *Oschr4-5* mutant, we examined the expression levels of 16 genes encoding enzymes involved in wax biosynthesis and three genes (*ROC4*, *WR1*, *WR2*) encoding transcription factors that activate the expression of wax biosynthetic genes [26,27,28,44,45]. Quantitative real-time polymerase chain reaction (RT-PCR) using RNA samples prepared from leaves of 30-day-old plants revealed that transcript levels of six wax biosynthetic genes (*GL1-4*, *WSL4*, *OsCER7*, *LACS2*, *LACS7*, *BDG)* and one transcription factor gene (*ROC4*) [26,27,28,44,45] were significantly upregulated in *Oschr4-5*, compared with WT (Figure 6A). This result suggests that loss of function of CHR4 affects the expression of important genes involved in the wax biosynthesis.

**Figure 7:**
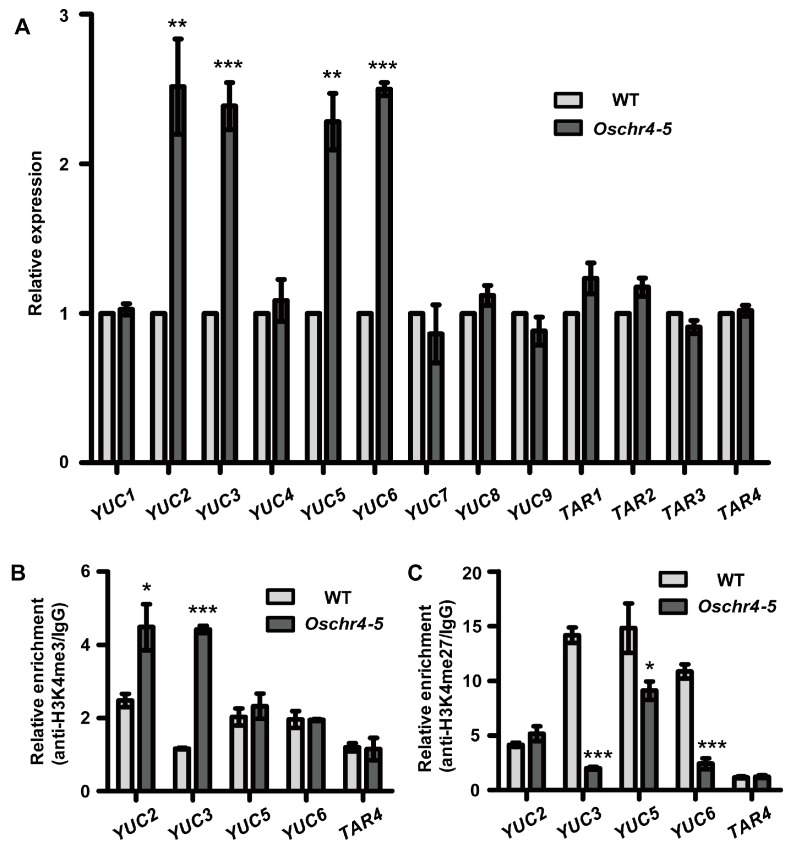
Expression and histone modification of genes involved in IAA biosynthesis in WT and *Oschr4-5*. (**A**) qRT-PCR analysis showed 4 of 13 tested IAA biosynthesis genes were up-regulated in 3-day-old seedlings of *Oschr4-5* relative to the WT. Data are the means ± standard errors (*n* = 3). Significance of data is tested by student’s *t* test. ** *p* < 0.01, *** *p* < 0.001. (**B**,**C**) ChIP-qPCR analysis showed variations of H3K4me3 (**B**) and H3K27me3 (**C**) levels on 4 up-regulated IAA genes in *Oschr4-5* compared to WT. Data are the means ± standard errors (*n* = 3). Significance of data is tested by student’s *t* test. * *p* < 0.05,*** *p* < 0.001.

The *YUCCA* gene, which encodes a flavin monooxygenase-like enzyme, is known to be involved in Trp-dependent auxin biosynthesis [46]. The *TAA*/*TAR* genes, which encode putative Trp aminotransferases, have been proposed to act in the same pathway with *YUCCA* genes [47]. To explain the increased IAA level in the *Oschr4-5* mutant, we measured the expression levels of 13 genes from the *YUCCA* and *TAA/TAR* family between three-day-old seedlings of WT and *Oschr4-5*. The transcript levels of four *YUCCA* related genes including *YUC2*, *YUC3*, *YUC5* and *YUC6* were upregulated significantly in the *Oschr4-5* mutants (Figure 7A). However, the other 9 genes showed no significant alteration in the *Oschr4-5* mutants. These results suggested that *OsCHR4* may influence the expression of several wax-related and IAA-related genes, and the increased gene expressions in the *Oschr4-5* mutants conduces to higher wax and IAA content in *Oschr4-5* than WT.

### 2.7. Histone Modifications of the Wax and Auxin Biosynthesis-Related Genes Were Affected in Oschr4-5

Histone modification is recognized as an important mechanism in the epigenetic regulation of gene expression. For example, trimethylation of histone H3 lysine 4 (H3K4me3) is generally correlated with gene activation, whereas trimethylation of histone H3 lysine 27 (H3K27me3) is generally correlated with gene repression [48,49]. As reported, the rice CHD3 protein OsCHR4/CHR729 is a chromatin remodeler and can modulate H3K4me3 and H3K27me3 level to regulate the genes expression [35]. In order to test whether increased expression of wax and auxin biosynthesis related genes in *Oschr4-5* could be attributed to altered histone modification, a ChIP analysis with antibody recognizing H3K4me3 and H3K27me3 was performed. Of the seven wax biosynthetic genes, three genes (*WSL4*, *CER7*, *BDG*) showed increased H3K4me3 and decreased H3K27me3 simultaneously, four genes showed either increased H3K4me3 (*GL1-4*, *LACS2*) or decreased H3K27me3 (*LACS7*, *ROC4*) (Figure 6B,C). Similarly, of the four auxin biosynthetic genes, one gene (*YUC3*) showed increased H3K4me3 and decreased H3K27me3 simultaneously, three genes showed either increased H3K4me3 (*YUC2*) or decreased H3K27me3 (*YUC5*, *YUC6*) (Figure 7B,C). *LACS1* and *TAR4,* two control genes which were not differentially expressed between *Oschr4-5* and WT, did not show significant changes in level of H3K4me3 and H3K27me3 (Figure 6; Figure 7). Taken together, *OsCHR4* affects modifications of histone protein associated with wax and auxin biosynthesis related genes and regulates their expression epigenetically.

## 3. Discussion

In rice, a total of six CHD-related (CHR) genes were found in its genome (http://www.chromdb.org), including *CHR705* (*Os07g46590*), *CHR702* (*PKL*, *Os06g08480*), *CHR703* (*Os01g65850*), *CHR4*/*CHR729* (*PKR1*, *Os07g31450*), *CHR723* (*Os06g01320*) and *CHR744* (*Os02g02050*). Among these CHR proteins, CHR729 was a typical chromatin remodeling factor which belonged to subfamily II (CHD3) [38]. It has been reported that three additional allelic mutants of *CHR4* displayed pleiotropic phenotypes [37,38,50]. In this study, three more allelic mutants of *CHR4* (*Oschr4-5, -6, -7*) were isolated, which exhibited similar pleiotropic phenotypes, including slight dwarfism, narrow and rolled leaves, and reduced chlorophyll contents in adaxial cells. More importantly, we found some novel phenotypes such as reduced number of longitudinal veins, increased IAA content, enhanced cuticular wax disposition and enhanced drought tolerance in *Oschr4-5*, which further illustrated the multiple functions of *OsCHR4* in plant development.

The closest homolog gene with *OsCHR4* in *Arabidopsis* is considered to be *AtCHR4 (PICKLE RELATED1, PKR1*) (Figure S2B), while their functions in plants seem to be different. Homozygous mutants of neither *PICKLE* homologs *PKR1* nor *PKR2* exhibited significant phenotypic differences to WT plants under standard growth conditions [51]. However, *OsCHR4* was revealed to be closely related with the development of rice leaves in this study. It is noteworthy that the function of *OsCHR4* is more similar to CHD3-related *PICKLE*. *PICKLE* was initially isolated from the *Arabidopsis pickle* mutant characterized by green tuberous root phenotype [52]. It has been reported that *PICKLE* functions in repression of ectopic stipules and meristems in leaf tissue and represses meristematic genes in carpel tissue [51,53]. *PICKLE* is not only involved in organ or cell polarity, but also plays an important role in cell differentiation [52]. Loss-of-function mutations in *PICKLE* could always influence cell transition from embryonic to vegetative development, thereby inducing pleiotropic phenotypes in the root, leaves and even the whole plants [54,55]. These phenotypes were just consistent with those of *Oschr4-5* isolated in this study and indicated a similar function between *OsCHR4* and *PICKLE.* However, T-DNA insertion mutation and RNAi of the closest homolog gene with *PICKLE* in rice (Os*CHR702*) did not produce any morphological phenotype [36]. Therefore, we suspect that the function of CHR proteins may be diversified between *Arabidopsis* and rice due to specific species difference.

Cuticular waxes are complex mixtures of very-long-chain fatty acids (VLCFAs) and their derivatives and provide effective protection for plants to confront various environmental stresses [28]. In this study, we discovered that *OsCHR4* negatively regulates the wax biosynthesis and more cuticle wax accumulated on the adaxial side of leaves in *Oschr4-5* mutant compared to the WT. Previous reports demonstrate that excessive deposition of cuticular wax can decrease nonstomatal water loss in plants [24,56]. We also proved that increased wax content in *Oschr4-5* results in reduced water loss and improved drought tolerance. As a result of increased wax deposition in *Oschr4-5*, the mutant *Oschr4-5* exhibits less chlorophyll leaching rate compared with WT. Furthermore, increased transcript levels of genes involved in wax biosynthesis in *Oschr4-5* were also in support of the regulation of *OsCHR4* in wax synthesis. Among the seven up-regulated genes in *Oschr4-5* (Figure 6A)*, WSL4* and its homolog gene *OsCER7* encode β-Ketoacyl-coenzyme A synthase (KCS) which is the key enzyme in the first step of VLCFA elongation [45,57]. *OsGL1-4* encodes the fatty aldehyde decarbonylase, which has been identified to positively regulate various steps of wax biosynthesis [21,22,23,24,25]. *LACS2* and *LACS7* encode long-chain acyl-CoA synthase that preferentially modifies VLCFAs for wax biosynthesis [58]. *BDG* (*BODYGUARD*) encodes an α/β-hydrolase fold protein important for cuticle development [28]. *ROC4* encodes a HD-ZIP IV transcription factor which positively regulates wax deposition in rice by activating wax biosynthetic gene such as *BDG* [28]. This result suggests multiple steps of wax biosynthesis pathway are affected by the loss of function of CHR4 and is consistent with the quantification data that many wax components were significantly increased in *Oschr4-5* (Figure 3C).

It has been demonstrated in previous research that *OsCHR4* is essential for various aspects of plant development via influencing the gibberellin pathway and ROS homeostasis [38,40]. In this study, we found that the content of IAA in *Oschr4-5* was significantly higher than that of WT. As reported, many narrow leaves genes were interrelated with IAA. Auxin overproduction is well known to lead to narrow and epinastic leaves. Ectopic overexpression of either *YUCCA* genes (encoding flavin monooxygenases) in *Arabidopsis* [46] or the Agrobacterium auxin biosynthetic gene *iaaM* (encoding tryptophan monooxygenase) and petunia *FLOOZY* gene (ortholog of *YUCCA*) in petunia resulted in narrow and rolled leaves with high levels of IAA [42,43]. In *Arabidopsis,* narrow and rolled leaves were also caused by elevated IAA levels by overexpressing cytochrome P450 *CYP79B2* which converts tryptophan (Trp) to indole-3-acetaldoxime [59]. A similar case was reported in rice, where *OsYUCCA8/NAL7* also affects leaf width via altered IAA content [6]. In this study, increased IAA content was also detected in the narrow leaf mutant *Oschr4-5*. Auxin is principally converted from tryptophan via the TAA family of aminotransfrases and the YUCCA (YUC) family of flavin-containing monooxygenases [60,61]. For further verification of the altered IAA concentration in *Oschr4-5*, we detected the expression of *TAA* and *YUC* family genes and found that expression levels of four *YUC* genes increased in *Oschr4-5* mutants compared with WT. The finding is consistent with increased IAA concentration in *Oschr4-5*, which strengthens the evidence that *OsCHR4* might regulate auxin biosynthetic process in rice. Interestingly, 31 *OsAux*/*IAA* genes involved in the auxin signaling pathway was significantly downregulated in *crl6,* an allelic mutant of *Oschr4-5* [39]. The decreased transcript level of *OsIAA* genes might be due to higher IAA content as revealed in this study. As early-response genes and negative regulators of auxin signaling, *Aux*/*IAA* transcripts tend to be rapidly induced and then repressed by elevated IAA content [62]. 

Histone modification was an important mechanism in regulation of DNA transcription and other activities. As reported, trimethylated histone H3 lysine 27 (H3K27me3) was associated with gene silencing and repression during plant development, while trimethylated histone H3 lysine 4 (H3K4me3) is generally correlated with gene activation [63,64]. In the *chr729* mutant, 56% and 23% of marked genes lost H3K27me3 and H3K4me3, while 754 and 724 genes gained ectopic H3K27me3 and H3K4me3 [36]. Meanwhile, *PICKLE* was revealed to be able to reduce the level of H3K27me3 on root stem cells, thereby promoting the expression of meristem marker genes [65]. To evaluate the histone modification states in the *Oschr4-5* mutant, ChIP was performed on several differently expressed genes involved in wax and auxin biosynthesis in this study. As a result, the increased level of H3K4me3 and/or reduced level of H3K27me3 in the *Oschr4-5* mutant were significantly correlated with increased expression of genes related to wax or IAA biosynthesis. Consistent with the genome-wide ChIP-Seq analysis showing that 724 genes were detected to have gained ectopic H3K4me3 in the *chr729* mutant [36], H3K4me3 was also increased in several auxin and wax biosynthesis genes in the *Oschr4-5* mutant in our study. The underlying mechanism for the increased H3K4me3 at some specific loci still remains elusive [36]. As a bifunctional chromatin regulator that recognizes and modulates both H3K4 and H3K27 methylation, the function of OsCHR4 might be determined by sequence-specific transcription factors that associates with their targets and/or by the recruitment of different histone modification enzymes that catalyze methylation or demethylation.

In conclusion, our results suggest that *OsCHR4* not only regulates the accumulation of cuticle wax on leaf surface, but also plays an essential role in the modulation of IAA biosynthesis. Expression levels of several genes involved in the two pathways were increased accompanied with up-regulated H3K4me3 or down-regulated H3K27me3. Epigenetic regulation by *OsCHR4* is required for multiple aspects of plant development.

## 4. Materials and Methods

### 4.1. Plant Materials and Growth Condition

The rice mutant *Oschr4-5* (requested from Dale Bumpers National Rice Research Center, Stuttgart, AR, USA) was isolated from the *indica* cultivar Indica 9 treated by gamma ray radiation. The other two allelic mutants *Oschr4-6* and *Oschr4-7* were isolated from the *japonica* cultivar Nipponbare treated with EMS in the Institute of Crop Science, Chinese Academy of Agricultural Sciences, Beijing, China. All rice plants were cultivated in the experimental field of Shandong Rice Research Institute (Jining, China) under natural growing conditions, and the climatic conditions during the rice growing season were listed in Table S2. The mutant phenotype inherited stably after continuous cultivation. Field data of the mutant and WT plants, including plant height, tiller number, leaf width and leaf length were measured at the heading stage. The transpiration rate of plants was detected in field using LI-6400XT Portable Photosynthesis System.

### 4.2. Microscopic Observation

At the heading stage, the top first leaf of the mutant and WT were cut into pieces (2 cm) in the widest part and divided into three groups. One group of samples were used for microscopic observation of leaf vein and transverse cross section. Another group of samples were air-pumped in 2% cellulase R-10 solution for 30 min and digested for at least 2 days. After washing with water, mesophyll cells were peeled and the abaxial epidermis was observed under microscope. The cell width was measured using the Image J software (https://imagej.nih.gov/ij/). Stomatal density (mm^−2^) was counted between two lateral veins in the center region of five individual leaves. Finally, the last group of samples were fixed in fixative solution (75% ethanol, 5% acetic acid, 5% glycerol, 5% formaldehyde and 10% deionized water) for at least one day, and then critical point dried, sputter-coated with gold and observed with a scanning electron microscope (UANTA 200).

### 4.3. Measurement of Chlorophyll and Indole Acetic Acid (IAA)

The top first leaf of plants at heading stage was used for chlorophyll measurement. Briefly, 0.05 g leaf samples were cut into small pieces and incubated in 80% acetone overnight in dark. Then the chlorophyll contents were measured and calculated by absorption spectrophotometry. For the measurement of IAA, 0.2 g freeze dried sample of the whole shoots of 3-day-old seedlings was harvested for each replicate. Free IAA extraction and measurement was performed as described previously [66].

### 4.4. Measurement of Cuticular Waxes

The content of cuticular waxes in the leaf of mutant and WT was measured as described previously [24]. Briefly, cuticular wax samples were first extracted from the top first leaf of plants at the heading stage by immersion in chloroform (60 °C, 30 s). A total of 20 µg n-tetracosane (C24, SUPELCO, Sigma, Saint Louis, USA) was added to these samples as an internal standard. Then the solutions were soaked with 100 mm^3^ bis-N,N-(trimethylsilyl) trifluoroacetamide (BSTFA, SUPELCO, Sigma) and 100 mm^3^ pyridine for 1 h at 70 °C. After evaporation and filtration (WHATMAN, PTFE, 13 mm × 0.22 μm), the compositions of cuticular waxes were analyzed using a capillary gas chromatograph equipped with an HP-1MS column (30 m length, inner diameter 0.32 mm, film thickness 0.25 µm) attached to a mass spectrometer (GCMS-QP2010, Kyoto, Japan). The GC-MS protocol included injection at 250, 50 °C for 2 min, ramped to 200 at 20 °C min^−1^, 2 min at 200 °C, ramped to 320 at 2 °C min^−1^, 14 min at 320 °C with supplied at 1.2 cm^3^ min^−1^ as the carrier gas. A flame ionization detector was used for quantitative analyses, and the quantification of each component was calculated by the equivalent ratio of mass to peak area between the component and internal standard.

### 4.5. Water Loss and Chlorophyll-Leaching Assays

Leaf water loss rate was measured using detached leaves of the same parts from 6 week-old seedlings cultured in climate chambers. Each leaf was kept in a box without lid, and weighed every 15 min [28]. The water loss rate was calculated by dividing the lost weight of leaf at different time points by the initial weight. In the assay of chlorophyll leaching, the leaves (0.25 g) were cut into 2 cm lengths and immersed in 25 cm^3^ 80% ethanol. 1 cm^3^ chlorophyll leaching solution was taken for spectrophotometry at wave lengths of 664 and 647 nm every 30 min. 1 cm^3^ 80% ethanol was used as control. The solution should be collected and poured back in the same tube after each measurement. The chlorophyll concentration was calculated by the formula of 7.939 A664 × 19.539 A647 [24]. The chlorophyll-leaching rate was given by dividing the concentration monitored at different time points by the chlorophyll concentration measured after 24 h of immersion. The experiment comprised three replicates.

### 4.6. Mapping and Sequencing

An F_2_ segregating population generated from a cross between *Oschr4-5* and 02428 (a polymorphic *japonica* cultivar) was used for mapping and cloning of the target gene. For the primary mapping, a DNA pool composed of DNA from 10 individual mutant plants was used for bulked segregation analysis (BSA) [67]. Approximately 200 polymorphic indel markers distributed evenly throughout the rice genome were used for the BSA analysis. For fine mapping, new indel markers were designed by utilizing genomic sequence information from *Indica* and *Japonica* (http://rgp.dna.affrc.go.jp/) (specific primers were shown in Table S3). DNA was extracted from fresh leaves of each plant. PCR program included 5 min at 95 °C followed by 35 cycles of 30 s at 94 °C, 30 s at 55 °C, 30 s at 72 °C, and a final extension of 10 min at 72 °C. PCR products were analyzed on a polyacrylamide gel stained with silver nitrate. The genomic region of the candidate gene was divided into several overlapping 1.5-kb fragments, and amplified using high-fidelity PrimeSTAR DNA polymerase (TaKaRa Bio, DaLian, China). The PCR program included 3 min at 94 °C followed by 40 cycles at 98 °C for 30 s, 55 °C for 5 s, 72 °C for 1.5 min, and a final extension at 72 °C for 10 min. PCR products were sequenced directly.

### 4.7. Real-Time Polymerase Chain Reaction (RT-PCR)

Total RNA was first extracted from rice plants with Trizol method, and then first-strand cDNA was synthesized using a Superscript III Reverse Transcription Kit (Invitrogen, Carlsbad, CA, USA). Semiquantitative PCR was performed using LA Taq DNA polymerase (TaKaRa) with the rice *ACTIN1* gene serving as an internal control. The PCR program included 1 min at 94 °C followed by 30 cycles at 94 °C for 30 s, 60 °C for 30 s, 72 °C for 30 s, and a final extension at 72 °C for 10 min. Quantitative PCR was performed using a SYBR Premix Ex Taq2 kit (TaKaRa) on ABI PRISM 7900HT under the following conditions: 10 s denaturing at 95 °C, 30 s annealing at 60 °C, 40 cycles. The mRNA amount relative to *ACTIN1* was finally calculated. Specific primers were shown in Table S4.

### 4.8. Sequence and Phylogenetic Analyses

Gene prediction was performed using online search software (http://rice.plantbiology.msu.edu/cgi-bin/gbrowse/rice). Exon/intron structures were identified by alignment of coding sequences (CDS) and genomic DNA sequences. Multiple sequence alignments were conducted using CLUSTALX software, and a phylogenetic tree was built using MEGA 4 software [68].

### 4.9. *Chromatin Immunoprecipitation (*ChIP*)* Analysis

The ChIP experiment was performed based on a previously published protocol [69]. Simply, 0.5 g of 3-day-old or four-leaf-old seedlings of *Oschr4-5* and WT were first harvested and crosslinked in 1% formaldehyde under vacuum for 15 min or 30 min, respectively. Then the isolated chromatin complex was fragmented to 200–500 bp by sonication, and 1% of the fragmented chromatin from each tube was kept as input DNA. The chromatin modification states of several target genes were analyzed using antibodies recognizing H3K4me3 (ab8580, Abcam, Cambridge, UK) and H3K27me3 (ab6002, Abcam) and the pre-immune serum (02-6502, Invitrogen) as a negative control. Then the immune complexes were collected with Protein A Agarose beads (P3476, Sigma). After reversing the crosslink, the precipitated and input DNAs were detected by real-time PCR, and the enrichment of H3K4me3 and H3K27me3 of each genes was quantified by normalizing the threshold cycle (*C*t) of the ChIP sample with that of the input with 2^(*C*t of input − *C*t of sample ChIP)^. Specific primers used in ChIP-PCR are shown in Table S4.

### 4.10. Accession Number

Sequence data from this article can be found in the GenBank databases under the following accession numbers: *OsCHR4* genomic DNA, MK765112; *OsCHR4* cDNA, MK765113.

## Supplementary Materials

Supplementary materials can be found at https://www.mdpi.com/1422-0067/20/10/2567/s1.

## Abbreviations

CHD3	Chromodomain Helicase DNA-binding (CHD) protein 3
ChIP	Chromatin immunoprecipitation
CHR4	CHD-related protein 4
NAL1	Narrow leaf 1
