# Supplementary material for: Mutations in the Rice OsCHR4 Gene, Encoding a CHD3 Family Chromatin Remodeler, Induce Narrow and Rolled Leaves with Increased Cuticular Wax
Source: Int J Mol Sci. 2019 May 25;20(10):2567. doi: 10.3390/ijms20102567 (PMC6566577; doi:10.3390/ijms20102567)
Supplement: Supplementary file 1 [file ijms-20-02567-s001.pdf]

Supplementary Materials

Mutations in the rice *OsCHR4* gene, encoding a CHD3 family chromatin remodeler, induce narrow and rolled leaves with increased cuticular wax

Tingting Guo[1, #], Daofeng Wang[2,#], Jingjing Fang[2], Jinfeng Zhao[2], Shoujiang Yuan[3], Langtao Xiao[1, *] and Xueyong Li[2, *]

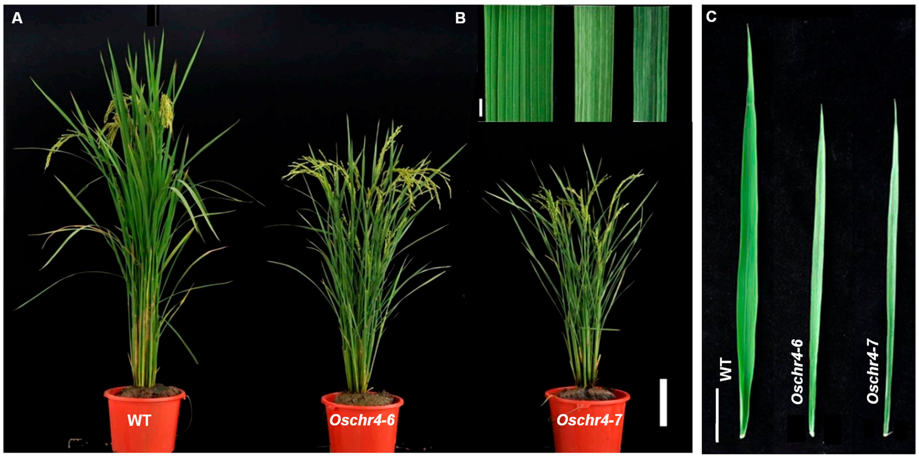

Figure S1. Phenotypic characterization of the allelic mutants *Oschr4-6* and *Oschr4-7*. (A) Phenotypes of wild type and mutants plants at mature stage. Bar = 15 cm. (B and C) Comparison of leaf length (B) and width (C) between wild type and mutants at mature stage. Bar = 0.2 cm (B), 3 cm (C).

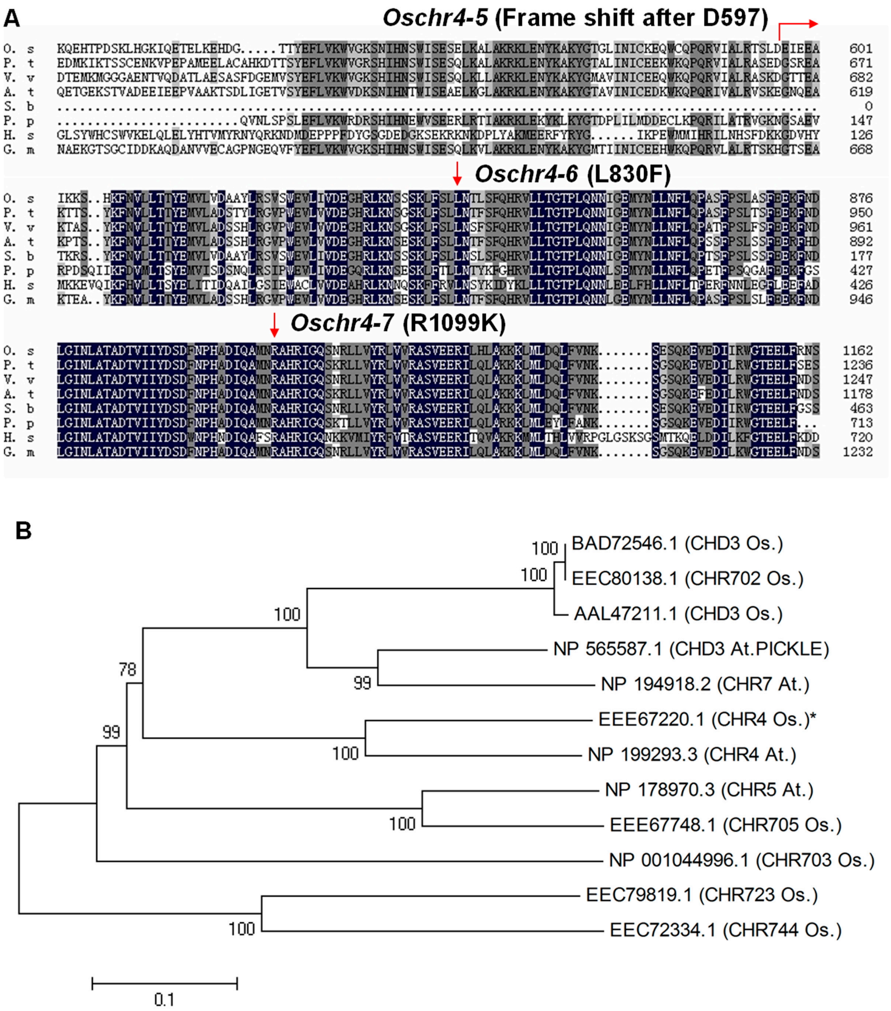

Figure S2. Alignment and phylogenetic analysis of the OsCHR4 proteins. (**A**) Multiple alignment of OsCHR4 amino acid sequence in eight species. The mutant sites were indicated as red arrows. (B) The evolutionary relationship among CHD families in rice and Arabidopsis.

Table S1. Field data of the mutant and Wild type rice plants

	Plant height (cm)	Tiller number	Leaf width (mm)	Leaf length (cm)
Indica cultivar Inidca 9 (Wild type)	99.22 ± 5.36	18 ± 2	18.29 ± 0.88	39.46 ± 5.41
Gsor 22 (*Oschr4-5*)	65.67 ± 3.73**	6 ± 1**	9.06 ± 0.54**	24.28 ± 2.63**
Japonica cultivar Nipponbare (Wild type)	103.50 ± 6.36	21 ± 3	15.70 ± 1.25	33.99 ± 3.48
S1-88 (*Oschr4-6*)	71.30 ± 6.19##	13 ± 2##	6.90 ± 0.26##	26.41 ± 1.77##
S2-16 (*Oschr4-7*)	80.41 ± 1.98##	13 ± 3##	9.00 ± 0.47##	25.33 ± 3.02##

Data were measured at the heading stage and represent as mean ± SE (n = 10). ** and ## represent significant different at $P < 0.01$ when compared with *Indica* cultivar Inidca 9 and *Japonica* cultivar Nipponbare, respectively.

Table S2. The climatic conditions of the experimental field

	Mean daily temperature	Mean precipitation
June	28 ℃	39 mm
July	33 ℃	76 mm
August	34 ℃	61 mm
September	32 ℃	30 mm
October	27 ℃	11 mm

Table S3. The primer sequences of markers used in mapping

marker	Forward primer	Reverse primer	Length (bp)[a]
ha1	GCGAACCGATAAAACTGCTC	AGAGGTGTATCAAAGCAATCGAG	104
ha2	GTGCCCGTAATGCTCTCAAT	TGAAAGGTTGATCCTGAACAT	149
ha3	CTCACCTAGACATTGTGCTT	GATAGGTTCTGAGTAGCCTC	195
ha4	TCACCTCTCAACTTAATCGA	AGTCCATCAAGCCATGATGC	241
ha5	AGACTGCTCATTTCTTGGGT	GAACACCTGTCACCTGATCA	242
ha6	AGTTCGTCCGGTTTTGATCG	GTAGAATAAGCGAAACAGCA	249
ha7	AAGTGCATATGCCAGCACAA	GTCCAACTTTAACCATCCGT	316
ha8	CAACAGCGTACATCCGAAAC	TGATCAAACCACACAGCCTA	254
ha9	TCGACCATCAGCGATTTGAC	TTTTCCATGCGCGGTGTTTG	180
ha10	TCCAATGGTGGTGGCTATGA	TGCCTGTCTAAGCGAGAGGT	225

Table S4. The primers used in RT-PCR and ChIP-qPCR

marker	Forward primer	Reverse primer
*CHR4*RT	CTCAGCGCCATGTGATTAAG	GATTCACCGAGACGTTTAGC
WSL4	ACTCAAGCCCAAGGACATCGACAT	GTTGCTGCGGAGCTTGTACTTGTT
CER7	ACCACCATCTGTGGTTGAGGACAA	TTGGCGATATAGCTTCCTGCGTCT
CER10	CGAGTGGTAACGGTGGCTAT	CTGTGTGGCAATGTTGAACC
LACS1	GGGAGTTACATACACCGATTTCG	CTTGAGGGCAGCAGTGACAA
LACS2	TGTGAAGGAGTCTGGAGGGTTGA	GCACCTGAAGACATAAGCCTCACA
LACS7	GTGGACGTGTGAGGCTTATGAC	GGAATTCCATTACATCAGCTGACA
LACS8	GGGAATGCGCTGGTTTTACA	GATCTCGAGGCACCCATCAG
ROC4	GGTGTATGGGCTGTAGTGGA	CCTTCGGCAGTTCATGTTT
BDG	GGCAAGATCGACAAATGCCTTGAG	GGCTAGTTCTTTCTGCCTTCTGAC
GL1-1	GTTCGTCTCGTCGATCCAAC	CTCATCTCTTTATGTATCCAAC
GL1-4	TCATCCTGCCGTCGCTG	CGGGAGAGGCTGATCCAGA
WR1	AGAAGTCCCACATTGGCGTGT	GCTCAGCAACTCCTCGATCATT
WR2	GCAACGCCAAGACCAACTTC	ACGCCAATGTGGGACTTCTC
WSL3	CAATTCTCGAGATGCCTCTATGT	TCTTGATGGACGCCATCTTC
FDH	TGCTGGCCAAGTCTGGCAT	TTGAAGAGGCTGCAGTTGACGA
KCR1	ACCCGCTCTACAGCGTCTAC	TACAGGGGTACCTGGCATTG
DWA1	GAAGACTGGGGCTGGGAAA	TGCGTAGATGCTAACGAGGTG
KCS2	GTACAGGTTTGGGAACACGTCG	GCTCAGTCGGTTGAAATCCTGG
YUC1	TCATCGGACGCCCTCAACGTCGC	GGCAGAGCAAGATTATCAGTC
YUC2	GTCCAAAGGGAGGAGTCGTCCAG	GCATGATGTTTACACCCGGCCTT
YUC3	CTGGTGACATCAAGGTACGG	ACTCCCGGTCCTTTAACCAG
YUC4	GCAGAATGGCCTGTACGCTGTTGG	CAGACCAGCACATGACGTGTCTAC
YUC5	ACCTCCTACGACGCCGCCATGATC	CTCCCAACACAGCGACGACAGAAC
YUC6	ACCGGATACCAAAGCAACGTC	GCAATGTCCTGTGCAACCTTAA
YUC7	AACACAGTGATCGCATGGACA	TCGAGGTAGTCGATGAACTGG
YUC8	ACTGTAGTGCATGCAAGAGGAGA	CCCAAGAACCGATGAGCTAAG
YUC9	CTGGCTCAAGAGTGATGACG	TCCTCGTAGCTGCCGTAGAT
TAR1	ATACTTCGGCGGTGACGACG	CGTCGGAGAAGTAGCTCATC
TAR2	CGCCCTACTACTCGTCATACC	GATTGTTCGGGGAGCAGAC
TAR3	GCTCCATGCAGCTCATCAAC	TCCCGGCCGTCAAACATGTC
TAR4	GGTGGCATCGCTTGAGCTAC	CCGTCGAACATGACGGTCTG
C-WSL4	ACTCCTCGTCATCTCGTCTC	CCGGCATGATCGATGCTCTC
C-CER7	TTGGGCTGAGAGTGAATGGG	TGAACTCGCGCTCGTTCACC
C-LACS2	AGCTGCGAATCTTCCCTTAG	GTTGCCTCGCATTGGTCTTG
C-LACS7	TTCCTACCCTTCCCGGTAAC	ATTTGTGGCGCGTGGGAAGC
C-ROC4	GACACGGAAACCCAGGTTTG	CTGGCCCGGGAGAAGATTAG
C-BDG	CCCGCCATCAATTCACTCTC	GTGGTGGTGGTCGTAGACTG
C-GL1-4	AGGCGAAGCAGTGGGTAGTG	CACGGCCATTCGGTCAAAGG

C-LACS1	CCCAATTGCGGTATCTCTTC	CAGGACTGCTCAAACAACTC
C-YUC2	CCCAAGTACCCAACACAATC	CAACTATTGGCCCTTGAACC
C-YUC3	CAAGTCTTCTGGCGATACAC	CTTGCCCTCAATTTCACTCC
C-YUC5	TAGGGTGTAGACGGCTGTAG	CCATCTTGTTGCGAGGTTGG
C-YUC6	GTGCACAGGCACAGCTACAC	AGCGAGAGCGAGGAAGTAAG
C-TAR4	AGCACGACGCGAAATCTAAC	GTGACCTATGCCATCCTATC
